# Application of PGF_2α_ at the moment of fixed-time artificial insemination in crossbred beef cows

**DOI:** 10.1590/1984-3143-AR2022-0012

**Published:** 2022-11-04

**Authors:** Jéssica Cristina dos Santos Marques, Gustavo Pereira Cadima, Ana Cláudia Fagundes Faria, Eduarda Arruda Guimarães, Fabiana Silva Oliveira, Ricarda Maria dos Santos

**Affiliations:** 1 Faculdade de Medicina Veterinária, Universidade Federal de Uberlândia, Uberlândia, MG, Brasil; 2 Faculty of Land and Food Systems, University of British Columbia, Vancouver, British Columbia, Canada

**Keywords:** FTAI, pregnancy, PGF_2α_, conception rate, beef cows

## Abstract

Although studies have shown positive effects of gonadotropin releasing hormone (GnRH) or prostaglandin F_2α_ (PGF_2α_) at the moment of fixed-time artificial insemination (FTAI) in the conception rate (CR) of cattle, its effects on treatments based on progesterone (P4) and estradiol benzoate (EB) is still not conclusive. The objective of this study was (1) to evaluate the effect of a PGF_2α_ analogue at FTAI in the CR of crossbred beef cows submitted to a 11d FTAI protocol based on P4 and EB; and (2) to describe the CR between PGF_2α_-treated and control cows in different body condition scores (BCS) and parity categories. Crossbred (½ Nellore and ½ Angus) beef cows were submitted to a synchronization protocol and randomly assigned into 2 groups: Control (n = 163), at FTAI cows received 2 mL of saline solution as a placebo, and PGF_2α_ (n = 163), at FTAI cows were treated with PGF_2α_ analogue (10 mg of dinoprost tromethamine). Pregnancy diagnosis was performed 33d post-FTAI. Binary logistic regression model was used to analyze the effect of PGF_2α_ treatment on CR. There was no difference in CR between PGF_2α_ and control groups (*P* > 0.05; odds ratio (OR) = 0.92; confidence interval (CI) = 0.59-1.4). A greater CR was found in heifers (*P* = 0.0006, OR = 2.65, CI = 1.61 - 4.38) and multiparous (*P* = 0.0006, OR = 2.12, CI = 1.04 - 4.3) when compared to primiparous cows. Cows with low BCS (4; 9-point scale) showed lower CR when compared with moderate BCS (5-6; 9-point scale) (*P* < 0.05; OR = 0.10; CI = 0.06 - 0.18). There was no numerical difference on CR between PGF_2α_-treated and control cows in different BCS and parity categories. The results suggested that the CR in this study was not influenced by 10 mg PGF_2α_ analogue at FTAI.

## Introduction

One of the major factors affecting profitability and productivity of bovine herds is reproductive performance (Binelli et al., 2006). In this context, biotechniques of animal reproduction, such as fixed-time artificial insemination (FTAI), have an important role in the cattle industry. The FTAI is characterized as a biotechnology that uses hormones treatments to synchronize estrus and ovulation of bovine females. It makes possible to predict ovulation time allowing artificial insemination (AI) without the need of estrous detection leading to increases in productive and reproductive performance of beef herds (Borges, 2007).

Several studies have evaluated different protocols of FTAI in cattle aiming to improve this biotechnology practice worldwide (Hiers et al., 2003; Gottschall et al., 2009; Meneghetti et al., 2009; Sales et al., 2011). In this context, prostaglandin F_2α_ (PGF_2α_) is one of the hormones that is largely used in estrous synchronization programs alone or in combination with progestins, gonadotropin releasing hormone (GnRH) and estrogens (Weems et al., 2006). Although, PGF_2α_ analogues are most used in FTAI protocols for its luteolytic functions, recent studies have shown that this hormone could be used at the moment of AI to improve conception rates (CR) in cattle (López-Gatius et al., 2004; Ambrose et al., 2015; Diniz et al., 2021).

Based on previous literature, administration of PGF_2α_ at the time of AI could potentially increase CR of bovine herds by its actions on uterine contractility (Stolla and Schmid, 1990), gamete transport (Hawk, 1983) and by the induction release of luteining hormone (LH) through independent mechanisms of luteolysis (Randel et al., 1996). Hawk (1983) reported that treatment with PGF_2α_ increased the amount of semen recovered from the female reproductive tract which might be linked to increases in the ability of sperm to enter the folds and cervices of the female tract due to increased muscle contractility (Hawk, 1983). In addition, increased frequency of LH release 6 h after PGF_2α_ treatment has been reported by Randel et al. (1996). Perhaps, PGF_2α_ at time of AI may also lead to increases in the pituitary responsiveness to GnRH and consequently enhance the release of LH leading to ovulation (Randel et al., 1996).

Using a FTAI protocol based on GnRH and progesterone (P4), Ambrose et al. (2015) reported that an injection of 10 mg of a PGF_2α_ analogue (dinoprost tromethamine) at time of AI led to increased CR in high producing dairy cows. Likewise, López-Gatius et al. (2004) demonstrated that a PGF_2α_ analogue (cloprostenol) given at AI increased the risk of pregnancy in primiparous lactating cows. In a recent study, Diniz et al. (2021) demonstrated that an injection of 12.5 mg of dinoprost tromethamine (PGF_2α_ analogue) at the time of AI tended to increase pregnancy rates of suckled Nellore cows that did not display estrous behavior. However, whether PGF_2α_ analogue at time the AI have impacts in the CR of crossbreed beef cows submitted to a FTAI protocol based on P4 and estradiol benzoate (EB) is still uncertain.

Furthermore, in lactating dairy cows the effects of PGF_2α_ analogue at the time of AI has been reported to be correlated with body condition score (BCS) and parity (Ambrose et al., 2015). Ambrose et al. (2015) demonstrated that PGF_2α_ treatment at time of AI increased CR of primiparous cows having low BCS. López-Gatius et al. (2004) reported a significant interaction between PGF_2α_ treatment and parity. In their study (López-Gatius et al., 2004), administration of PGF_2α_ analogue at time of AI increased pregnancy rates of primiparous repeat breeder cows. However, the interactions between PGF_2α_ treatment at FTAI and BCS and parity in crossbreed beef cows still unknown.

Therefore, the first objective of this study was to evaluate the effect of 10 mg of PGF_2α_ analogue at the moment of FTAI in the CR of crossbred beef cows submitted to the FTAI protocol based on P4 and EB. Our second objective was to describe the CR of PGF_2α_-treated and control cows classified in different BCS and parity categories. We hypothesized that (1) cows treated with 10 mg of PGF_2α_ analogue at the moment of FTAI will have greater CR in comparison to cows receiving 2 mL of saline solution as a placebo, (2) there will be numerical differences in CR between PGF_2α_-treated and control cows classified in different BCS and parity categories.

## Methods

This study was conducted between November 2017 and March 2018 in a commercial beef cattle farm in Mato Grosso, Brazil. All experimental procedures were approved by the Animal Care Committee of the Federal University of Uberlandia, Uberlandia - MG Brazil, following the protocol # 016/13.

### Animals, housing, and management

The commercial herd consisted of crossbred (½ Nellore and ½ Angus) beef cows housed in grazing systems. The animals were maintained in continuous grazing with *Panicum maximum* cv. Massai, supplemented with mineralized salt and water *ad libitum* intake throughout experiment time. Cows were housed in lots of approximately 40 animals. The farm was located in Mato Grosso, region of tropical climate, with an annual average temperature of 30 °C, rainfall index varying from 1,800 to 2,200 mm per year, and the concentration of rain varying from September to April (Pereira et al., 2020). Health management consisted in vaccinations against foot and mouth disease and brucellosis, as well as periodic administration of antiparasitic agents.

### Experimental design

The inclusion criteria was performed based on the assessment of health status, days postpartum greater than 30 days, absence of uterine disease and BCS of at least 4 points on the scale proposed by Wagner et al. (1988). The scale of BCS used in this study (Wagner et al., 1988) ranged from 1 = very thin to 9 = obese and scores were assessed at the time of enrolment by a trained technician. Cows were organized in breeding lots with equal distribution of BCS, parity and treatment.

### Synchronization protocol and ultrasonographic analysis

All cows enrolled in this study were submitted to a synchronization protocol and randomly assigned into two groups at FTAI time. The FTAI protocol were as follows: D-11 intramuscular (i.m) injection of 2.0 mg of EB (Sincrodiol^®^ Ouro Fino) and insertion of the intravaginal device containing 1.0 g of P4 (Sincrogest ^®^ Ouro fino); D-4 12.5 mg (i.m) of dinoprost tromethamine (PG, Lutalyse^®^, Zoetis); D-2 removal of the intravaginal P4 device and 1.0 mg (i.m) of estradiol cypionate (E.C.P.^®^, Zoetis) and D0 fixed-time AI with frozen-thawed semen in all animals. Semen of different bulls (n = 3) with similar fertility were used in this study and were equally distributed within breeding lots, parity and BCS categories.

On D0 cows were randomly assigned into two groups: Control Group (n = 163), which at the moment of FTAI received 2 mL of saline solution as a placebo and PGF_2α_ Group (n = 163), which was treated at the moment of FTAI with PGF_2α_ analogue (10 mg of dinoprost tromethamine, Lutalyse^®^ Zoetis).

**Figure 1:**
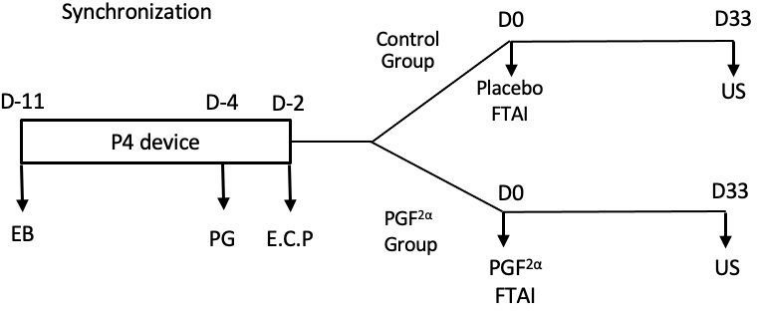
Experimental timeline with synchronization protocol, treatment groups and ultrasono**graphic ana**lysis. EB = estradiol benzoate, P4 device = intravaginal progesterone (P4) device, PG = dinoprost tromethamine, E.C.P = estradiol cypionate, FTAI = fixed-timed artificial insemination, PGF_2α_ = dinoprost tromethamine, US = ultrasound examination.

Pregnancy diagnosis was performed on average of 33 days after FTAI by ultrasound examination (US, Mindray DP3300®, Shenzhen, China), using a 7.5-MHz linear rectal transducer, for the detection of an embryonic vesicle with a viable embryo. Figure 1 summarizes the experimental timeline.

### Statistical analysis

Statistical analyses were carried out using SAS Studio (version 3.8, SAS Institute Inc., Cary, NC). Descriptive analyses were performed using PROC UNIVARIATE procedure and data were verified for presence of outliers using graphical assessment. BCS was used to categorize cows as moderate BCS (> 5 to 6) and low BCS (= 4) using a 9-point scale proposed by Wagner et al. (1988). Binary logistic regression model was used to analyze the effect of PGF_2α_ treatment on CR using PROC GLIMMIX procedure with cow as experimental unit and breeding lot as random effect. BCS categories, parity and bull were tested (fixed effects) as possible confounders and kept in the models if statistically significant (*P* ≤ 0.2). Frequency distributions of CR between treatment groups, bull, parity and BCS categories were performed using PROC FREQ procedure. Results from logistic regression models are presented as odds ratios (OR) and statistical significance was established as *P-*value ≤ 0.05.

## Results

A total of 326 crossbreed beef cows (168 heifers, 111 primiparous and 47 multiparous) were enrolled into the study. Cows had an average BCS of 4.62 (9-point scale) and the overall CR was 62.27%.

CR was similar between cows in the control and PGF_2α_ groups (*P* > 0.05; odds ratio (OR) = 0.92; confidence interval (CI) = 0.59 - 1.4). Table 1 summarizes the frequency distribution of CR between experimental groups.

**Table 1:** Effect of PGF_2α_ treatment at the time of fixed-time artificial insemination (FTAI) in the conception rate of crossbred beef cows submitted to a FTAI protocol based on progesterone (P4) and estradiol benzoate (EB).

**Group**	**n**	**Conception rate (%)**	***P*-value**
PGF_2α_	103/163	63.1	0.757
Control	100/163	61.3	

Semen of different bulls did not affect the CR in this study (*P* > 0.05; OR = 0.86; CI = 0.43 - 1.71). Table 2 summarizes the frequency distribution of the CR between the bulls used at AI.

**Table 2:** Effect of the bull used in the fixed-time artificial insemination (FTAI) protocol on the conception rate of crossbred beef cows submitted to a FTAI protocol based on progesterone (P4) and estradiol benzoate (EB).

**Bull**	**n**		**Conception rate (%)**	***P*-value**	
Bull 1		87/144	60.4		0.492
Bull 2		86/135	63.7		
Bull 3		30/47	63.8		

**Figure 2:**
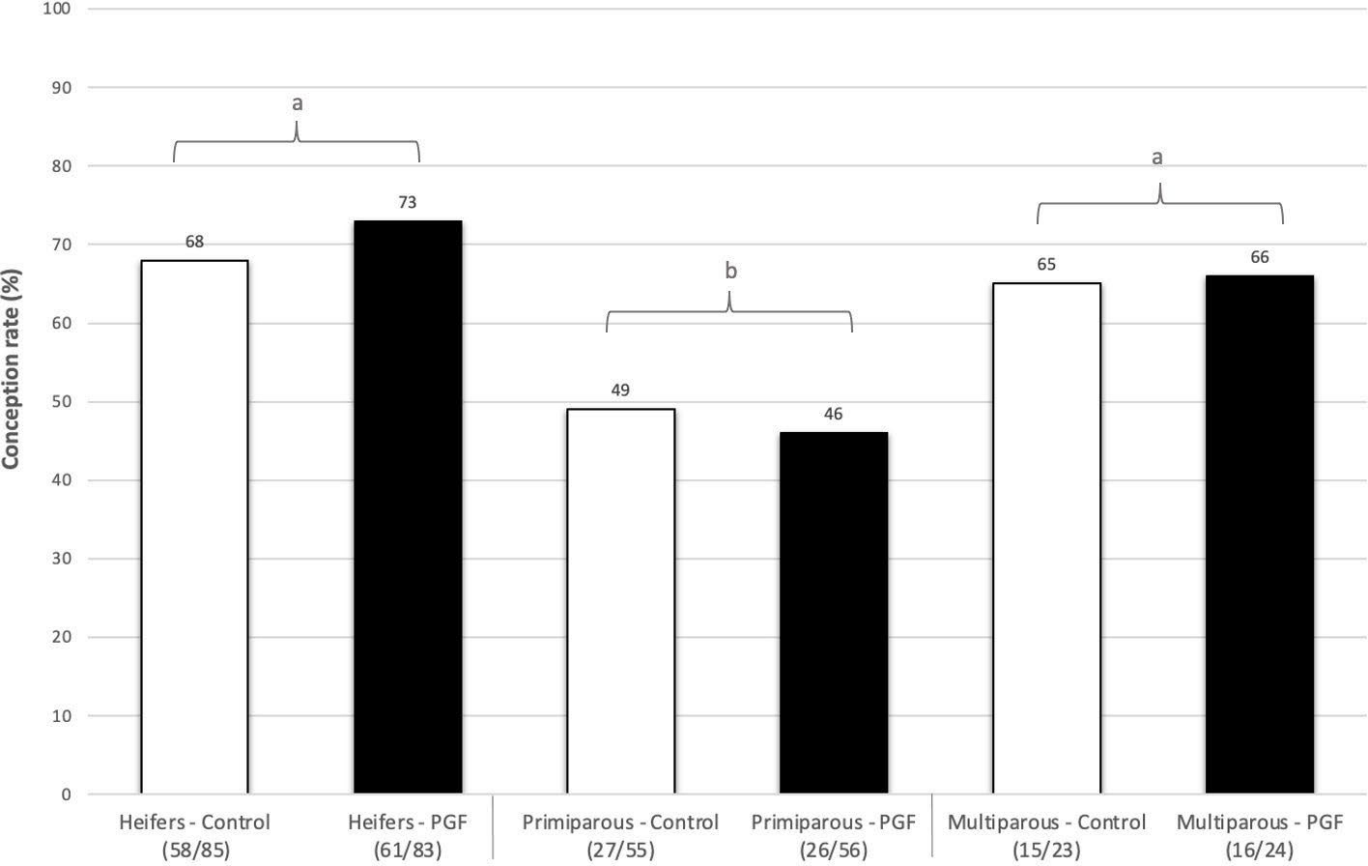
Frequency distribution of conception rate between experimental groups (PGF_2α_ vs. control) and parity categories (Heifers vs. Primiparous vs. Multiparous). Cows assigned in the group control received 2 mL of saline solution as a placebo whereas cows assigned to PGF_2α_ group received 10 mg of dinoprost tromethamine at the moment of FTAI. a,b: different letters indicate difference between variables (*P* < 0.05).

A greater CR was found in heifers and multiparous cows when compared to the primiparous cows (*P* < 0.05). The odds of pregnancy was 2.12 times greater for multiparous cows (*P* = 0.0006, CI = 1.04 - 4.3) and 2.65 times greater for heifers (*P* = 0.0006, CI = 1.61 - 4.38) when compared to primiparous cows. However, there was no numerical difference in CR between PGF_2α-_treated and control cows classified in different parity categories. Figure 2 summarizes the frequency distribution of CR between the treatment groups and parity categories.

**Figure 3:**
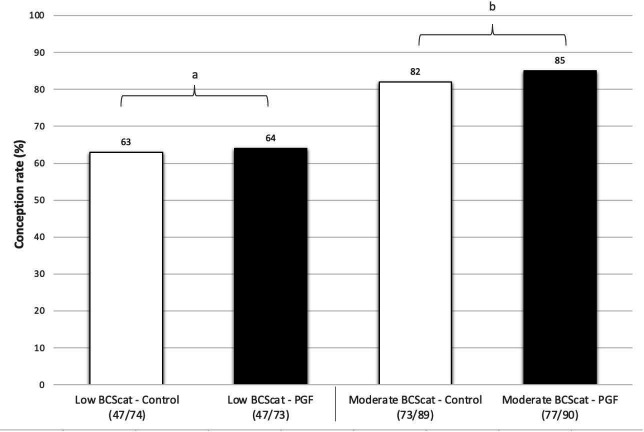
Frequency distribution of conception rate between body condition score (BCS) categories (moderate BCS vs. low BCS) and experimental groups (PGF_2α_ vs. control). BCS was used to categorize cows as moderate BCS (5 to 6) and low BCS (= 4) using a 9-point scale proposed by Wagner et al. (1988). Cows assigned in the group control received 2 mL of saline solution as a placebo whereas cows assigned to PGF_2α_ group received 10 mg of dinoprost tromethamine at the moment of FTAI. a,b: different letters indicate difference between variables (*P* < 0.05).

Cows categorized as low BCS (= 4) had lower CR when compared to cows categorized as moderate BCS (> 5 to 6) (*P* < 0.05; OR = 0.10; CI = 0.06 - 0.18). However, there was no numerical difference in CR between PGF_2α-_treated and control cows classified in different BCS categories Figure 3 summarizes the frequency distribution of CR between the treatment groups and BCS categories.

## Discussion

The main objective of this study was to evaluate the application of 10 mg of PGF_2α_ analogue at the moment of FTAI in crossbred beef cows. The second objective of this study was to describe the CR of PGF_2α_-treated and control cows classified in different BCS and parity categories. Our findings demonstrated that PGF_2α_ treatment at FTAI time did not affect CR, primiparous cows had lower CR when compared to heifers and multiparous cows, and moderate BCS cows had increased CR in comparison to low BCS cows. Furthermore, we demonstrated that there was no numerical difference in CR between PGF_2α_-treated and control cows classified in different BCS or parity categories.

In this study, CR was not different between cows receiving placebo solution or 10 mg of PGF_2α_ analogue at AI time. A variety of studies have reported the effect of PGF_2α_ treatment in dairy cows (Archbald et al., 1992; López-Gatius et al., 2004; Gabriel et al., 2011; Ambrose et al., 2015). Gabriel et al. (2011) evaluated the effect of a PGF_2α_ analogue at AI in spontaneous episodes of estrus of dairy cows. Similarly to our results, Gabriel et al. (2011) reported no effect of 25 mg of PGF_2α_ analogue (dinoprost) intramuscular at AI in pregnancy rates. However, when testing the deposition of 0.5 mg/mL of PGF_2α_ analogue solution in the uterine lumen immediately after AI a 12% increase in pregnancy rate was reported. Likewise, Ambrose et al. (2015) evaluated the effect of two different doses of PGF_2α_ at FTAI, using a synchronization protocol based on GnRH and P4, in the CR of dairy cows. Interestingly, in the results reported by Ambrose et al. (2015) the application of 5 mg of PGF_2α_ analogue (dinoprost tromethamine) at AI did not affect CR whereas 10 mg of PGF_2α_ analogue at AI was shown to increase the CR of dairy cows. Furthermore, López-Gatius et al. (2004) demonstrated that administration of a PGF_2α_ analogue (cloprostenol) given at AI increased the risk of pregnancy in 1.9 times. Perhaps the lack of effect presented in our results may be due to the use of different synchronization protocols as well as the use of beef cows instead of high producing dairy cows, different doses and different PGF_2α_ analogues. Evaluating the use of different synchronization protocols, Bridges et al. (2014) demonstrated differences in the diameter of the ovulatory follicles, different estradiol concentrations as well as different P4 concentrations in the ensuing luteal phase comparing a 7-day and a 5-day synchronization program in primiparous beef cows. In addition, greater negative energy balance in cows intensively selected for milk production has been linked with negative effects in pulsatile secretion of LH and reduced follicular responsiveness to LH and FSH (Diskin et al., 2003). Intra-follicular PGF_2α_ has been reported to play a critical role in the overall steroidogenic LH effect and in the follicular breakdown shortly before ovulation (Hunter, 2003). In addition, PGF_2α_ concentrations was shown to remain at baseline in follicles that fail to ovulate (Hunter, 2003). Therewith, it is possible to hypothesize that the effects of PGF_2α_ at time of AI in the CR may only be useful in cows which stress factors can affect ovulation such as high producing dairy cows with reduced secretion of LH and reduced follicular responsiveness to LH.

Reproductive traits such as age at puberty (Pitchford et al., 2022), as well as CR (Osoro and Wright, 1992) were reported to differ between purebred and crossbreed beef cows. Although several studies have evaluated the effect of PGF_2α_ analogues in the CR of beef cows (Leonardi et al., 2012; Pfeifer et al., 2018; Noronha et al., 2020), to the best of our knowledge this is the first study evaluating the effect of 10 mg of PGF_2α_ analogue at the moment of AI in crossbreed (½ Nellore and ½ Angus) beef cows. In Nellore cows, Noronha et al. (2020) assessed the effect of an extra injection of PGF_2α_ analogue (dinoprost tromethamine) 2d before AI during a FTAI protocol. In their study (Noronha et al., 2020), cows receiving an extra injection of PGF_2α_ had greater pregnancy per FTAI and larger preovulatory follicle in comparison to control cows. Diniz et al. (2021) also evaluated the effect of dinoprost tromethamine (PGF_2α_ analogue) at the time of AI, using a dose of 12.5 mg, in Nellore cows and demonstrated that PGF_2α_ treatment tended to increase pregnancy rates of suckled Nellore cows that did not display estrous behavior. Pfeifer et al. (2018) evaluated the effect of an extra injection of PGF2α, via intramuscular or subcutaneous, in the 9th day of a FTAI protocol in Nellore cows. Similarly to our study, no difference was found in the CR of cows treated with PGF2α analogue (cloprostenol) and control cows. However, besides the lack of effect in the CR, Pfeifer et al. (2018) reported higher ovulation rates in cows treated with PGF_2α_ when compared to cows treated with estradiol cypionate. Likewise, Leonardi et al. (2012) reported positive effects of PGF_2α_ treatment in ovulation rates of prepubertal crossbred Angus heifers whether or not exogenous P4 was used as a pre-treatment. Given the studies mentioned above, it seems that PGF_2α_ at time of AI may have the potential to improve fertility outcomes in Nellore beef cows. However, the lack of effect of PGF_2α_ treatment presented in the current study may indicate that 10 mg PGF_2α_ analogue at time of AI does not impact the CR of crossbred beef cows. Although our study does not include ovulation measurements, the fact that PGF_2α_ treatment was shown to affect ovulation rates (Pfeifer et al., 2018; Leonardi et al., 2012) as well as pregnancy rates of Nellore cows that did not display estrous behavior (Diniz et al., 2021) may emphasize (1) the auxiliary function of PGF_2α_ in the induction of LH release in Nellore cows and (2) the positive effects of PGF_2α_ treatment on CR of Nellore cows under factors that affect ovulation such as lack of estrous expression. The expression of estrus has been positively associated with ovulation and pregnancy rates in several studies (Burnett et al., 2018; Madureira et al., 2015 , 2019). Future research is needed to evaluate whether PGF_2α_ treatment affect ovulation in crossbreed beef cows. In addition, it is noteworthy to emphasize that the sample size used in our study may not fully reflect the beneficial effects of PGF_2α_ treatment in the CR of crossbred beef cows.

The variable bull did not affect the CR obtained in this study. In FTAI programs, the use of different bulls can be positively or negatively associated with fertility results (Sá, 2012). According to Sá (2012), it is evident that there is an individual effect on the fertility of the bulls, as well as a variation between samples of semen collected from the same bull. A data survey using more than 7 thousand inseminations performed in Mato Grosso, Brazil reported by Sá (2012) demonstrated significant differences in CR between samples of semen collected from the same bull in different days. In addition, the effect of the bull on conception may differ depending on the seasons of the year as well as regarding to the parity of the females (Luz et al., 2018). Luz et al. (2018) reported a difference of more than 20% in CR when comparing the same bull in different seasons of the year. Although, this study has selected bulls with high fertility to avoid low fertility results (Sá, 2012) and bias in our research, the monitoring of this variable is necessary for accurate evaluation of the PGF_2α_ treatment effect at FTAI on fertility.

Parity affected the CR observed in our study. Primiparous cows showed lower CR when compared to multiparous and heifers, however, there was no numerical difference in CR between PGF_2α-_treated and control cows classified in different parity categories. Likewise, other authors have reported primiparous cows having the worst reproductive performance (López-Gatius et al., 2004; Gabriel et al., 2011; Baruselli et al., 2012; Grillo et al., 2015; Martins et al., 2017). Gabriel et al. (2011) reported first parity cows having lower pregnancy rates when cows were pooled across the PGF_2α_ and saline groups. Oppositely, in the study of Ambrose et al. (2015) primiparous cows had greater CR in comparison to multiparous cows. Interestingly, in their study (Ambrose et al., 2015) a interaction was found between PGF_2α_ treatment and parity. Primiparous cows with low BCS tented to have improved CR when treated with PGF_2α_ analogue. The interaction between PGF_2α_ treatment and parity was also reported by López-Gatius et al. (2004). In their study (López-Gatius et al., 2004), primiparous cows treated with PGF_2α_ analogue showed 3.6 greater odds of pregnancy in comparison to not treated primiparous cows. In the study of Pfeifer et al. (2018), evaluating the effect of PGF_2α_ treatment in Nellore beef cows, parity effects were no reported to allow comparison with our study. Because primiparous beef cows were reported to experience pronounced negative energy balance due to increased energy demand for pregnancy, lactation as well as their growth (Wathes et al., 2007), we expected this animal category to have lower fertility results. In addition, given the positive effect of PGF_2α_ treatment in cows which stress factors can affect ovulation (Ambrose et al., 2015; Diniz et al., 2021), we expected to notice numerical differences in CR between PGF_2α_-treated and control primiparous cows. Although, parity was shown to affect the CR in this study, it is not clear why there was no numerical differences in CR between PGF_2α_-treated and control primiparous cows. Thus, our results may indicate that the effects of PGF_2α_ analogue at time of AI does not impact the CR of crossbreed beef cows classified in different parity categories.

BCS affected the CR observed in this study. Low BCS cows had lower CR when compared to moderate BCS cows. However, there was no numerical difference in CR between PGF_2α-_treated and control cows classified in different BCS categories. It is well known that animals having adequate BCS (5 to 6, 9-point scale), are more likely to conceive in a shorter period of time post-partum, have reduced calving to conceive interval and have increased reproductive efficiency (Sales et al., 2011; Sá et al., 2013). The energy restriction can directly influence the hypothalamus, promoting an increase in the negative feedback of the estradiol, reducing the frequency of LH release due to the lower energy intake (Imakawa et al., 1987). Although, several studies have reported low BCS cows having greater risk of low CR (Pryce et al., 2001; Ambrose et al., 2015; Pfeifer et al., 2018); Ambrose et al. (2015) demonstrated that dairy cows having lower BCS were affected positively by PGF_2α_ at the moment of FTAI. Lower BCS cows had greater CR when treated with PGF_2α_ analogue at FTAI when compared to control cows (Ambrose et al., 2015). Pfeifer et al. (2018) also reported that the probability of pregnancy increased with the increasing BCS in beef cows, however no interaction between PGF_2α_ treatment and BCS were reported. Given the positive effects of PGF_2α_ treatment in the CR of cows under stress factors that can potentially affect ovulation (Ambrose et al., 2015; López-Gatius et al., 2004), we expected to see a numerical difference between PGF_2α_-treated and control cows classified in different BCS categories. It is also not clear why there was no numerical differences between PGF_2α_-treated and control cows having different BCS. Thus, our results may suggest that administration of PGF_2α_ analogue at time of AI does not affect CR of crossbreed beef cows classified in different BCS categories.

## Conclusions

In conclusion, the application of a PGF_2α_ analogue at the moment of FTAI in a protocol based on P4 and EB did not result in greater CR in crossbred beef cows. However, it was observed that moderate BCS cows had greater CR in comparison to low BCS cows and primiparous cows had lower CR in comparison to heifers and multiparous cows. No numerical differences were found in PGF_2α_-treated and control classified in different parity and BCS categories.
